# Worldwide Protein Data Bank validation information: usage and trends

**DOI:** 10.1107/S2059798318003303

**Published:** 2018-03-02

**Authors:** Oliver S. Smart, Vladimír Horský, Swanand Gore, Radka Svobodová Vařeková, Veronika Bendová, Gerard J. Kleywegt, Sameer Velankar

**Affiliations:** a Protein Data Bank in Europe, European Molecular Biology Laboratory, European Bioinformatics Institute, Wellcome Genome Campus, Hinxton, Cambridge CB10 1SD, England; b Faculty of Science, National Centre for Biomolecular Research, Kamenice 5, 625 00 Brno, Czech Republic; c CEITEC – Central European Institute of Technology, Masaryk University, Kamenice 5, 625 00 Brno, Czech Republic; d National Centre for Biomolecular Research, Faculty of Science, Masaryk University, Kamenice 5, 625 00 Brno, Czech Republic; e Institute of Mathematics and Statistics, Masaryk University, Kotlářská 2, 611 37 Brno, Czech Republic

**Keywords:** PDB, Protein Data Bank, three-dimensional macromolecular structure, validation, quality control, X-ray crystallography

## Abstract

Realising the importance of assessing the quality of the biomolecular structures deposited in the Protein Data Bank (PDB), the Worldwide Protein Data Bank (wwPDB) partners established Validation Task Forces to obtain advice on the methods and standards to be used to validate structures determined by X-ray crystallography, nuclear magnetic resonance spectroscopy and three-dimensional electron cryo-microscopy. The resulting wwPDB validation pipeline is an integral part of the wwPDB OneDep deposition, biocuration and validation system. The wwPDB Validation Service webserver (https://validate.wwpdb.org) can be used to perform checks prior to deposition. Here, it is shown how validation metrics can be combined to produce an overall score that allows the ranking of macromolecular structures and domains in search results. The ValTrends^DB^ database provides users with a convenient way to access and analyse validation information and other properties of X-ray crystal structures in the PDB, including investigating trends in and correlations between different structure properties and validation metrics.

## Abstract 2

The use of validation metrics to rank macromolecular structures, as well as a web tool to investigate trends in and correlations between different properties and validation metrics, are described.

## 1. Introduction   

The Protein Data Bank is the single global archive of experimentally determined three-dimensional structures of biological macromolecules. The PDB archive was established in 1971 (Protein Data Bank, 1971 ▸) and archives coordinate models and experimental data for macromolecules determined by X-ray crystallography, nuclear magnetic resonance spectroscopy (NMR), three-dimensional electron cryo-microscopy (3DEM) and other structure-determination techniques. The PDB archive is managed by an international consortium, the Worldwide Protein Data Bank (wwPDB; https://wwpdb.org; Berman *et al.*, 2003 ▸), which has four partners: the Research Collaboratory for Structural Bioinformatics (RCSB PDB; Berman *et al.*, 2000 ▸), the Biological Magnetic Resonance Data Bank (BMRB; Ulrich *et al.*, 2008 ▸), Protein Data Bank Japan (PDBj; Kinjo *et al.*, 2017 ▸) and the Protein Data Bank in Europe (PDBe; Velankar *et al.*, 2016 ▸). The wwPDB collaboration was established in 2003 and the partner sites manage the deposition and annotation of biomacromolecular structures and release data into the PDB archive each week. The wwPDB partner sites collaborate on ‘data-in’ activities, including deposition and annotation software and policies to ensure the availability of a uniform archive. The deposition and annotation of macromolecular structure data for the PDB and the Electron Microscopy Data Bank (EMDB; Tagari *et al.*, 2002 ▸; Lawson *et al.*, 2011 ▸), which archives the electric potential maps from 3DEM experiments, is managed using the recently launched OneDep system (Young *et al.*, 2017 ▸).

The wwPDB organization works closely with the structural biology community (including journals and funding agencies) to ensure that experimental data accompany the coordinate models deposited in the PDB. Since 2008, X-ray crystal structure depositions to the PDB have required the deposition of the underpinning structure-factor data. Similarly, since 2010, the deposition of atomic models obtained by NMR has required the deposition of restraint and chemical shift information. Since August 2016, the deposition of electric potential maps in the EMDB has also been mandatory for atomic coordinate models obtained using 3DEM and deposited in the PDB.

As of 7 February 2018, the PDB archive contains 137 478 structures of biological macromolecules and complexes, of which 123 125 were determined using X-ray crystallography, 12 159 using NMR and 1793 using 3DEM, with the remaining 401 structures having been determined using either another structure-determination method or a combination of methods. These data are widely used by the structural biology community itself, but the wider biomedical community has also been making extensive use of PDB data for many years (Bousfield *et al.*, 2016 ▸). The PDB archive is also used by more than 200 different life-science data resources (Shao *et al.*, 2017 ▸) and thus supports a wide range of biomedical research.

One of the challenges in exploiting the macromolecular structure data archived in the PDB is assessing the quality of the coordinate model and the supporting evidence provided by the experimental data. To address this issue and to allow systematic assessment of the quality of the macromolecular structure data in the PDB, the wwPDB partners have established method-specific Validation Task Forces (VTFs). These VTFs consist of experts from the community who provide recommendations on assessing the quality of both the coordinate model and the supporting experimental evidence for each experimental method. The three VTFs for crystallo­graphy (Read *et al.*, 2011 ▸), NMR (Montelione *et al.*, 2013 ▸) and 3DEM (Henderson *et al.*, 2012 ▸), which was established in collaboration with the EMDB project partners (Lawson *et al.*, 2011 ▸), have provided recommendations which have now been implemented in the wwPDB validation pipeline (Gore *et al.*, 2012 ▸, 2017 ▸). The introduction of this pipeline, and the wwPDB validation report (VR) files that it produces (in both human-readable and machine-readable formats), has significantly expanded the scope of quality assessment provided by the wwPDB partners. Apart from the standard geometric validation of bond lengths and angles, Ramachandran analysis and close contacts, the validation pipeline incorporates community standards for assessing side-chain conformations, clashscore assessment using *MolProbity* (Chen *et al.*, 2010 ▸), assessment of experimental X-ray data using *phenix.xtriage* (Zwart *et al.*, 2005 ▸) and assessment of data–model agreement using the EDS (Kleywegt *et al.*, 2004 ▸) for X-ray structures. The VRs also include an assessment of nucleic acid and carbohydrate structures, although these are still rudimentary. Furthermore, the VR includes geometric assessment of small-molecule ligand structures using *Mogul* (Bruno *et al.*, 1997 ▸), which compares the geometry of a small molecule with a large set of high-quality small-molecule crystal structures available in the Cambridge Structural Database (CSD; Groom *et al.*, 2016 ▸). The experimental evidence that supports the presence, location, orientation and conformation of small-molecule ligands is also assessed by electron-density-fit metrics.

**Figure 1:**
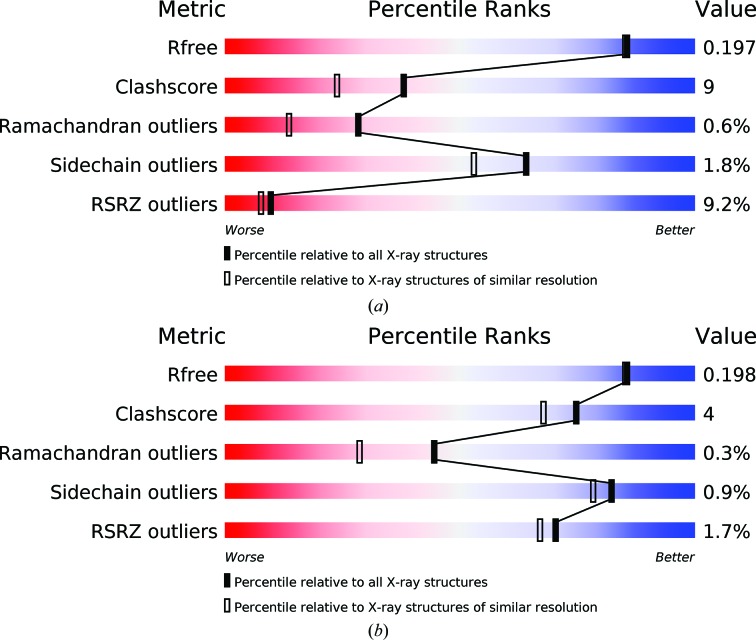
Slider plots for PDB entries 2de2 (*a*) and 2de4 (*b*).

One of the goals of developing the validation reports was to help scientists who are not necessarily experts in structural biology to assess the quality of any given structure. The ‘slider plot’ (Fig. 1 ▸) recommended by the X-ray VTF (Read *et al.*, 2011 ▸) provides a simple depiction of several key quality metrics of a given entry with respect to the whole archive and also in comparison to other entries at similar resolution (in the case of X-ray structures) or to other entries determined using the same technique (for NMR and 3DEM structures). The slider plot shows the value of each metric mapped to a percentile score, with the best entries (high percentile scores) shown at the ‘blue end’ of the slider, while entries with lower percentile scores appear near the ‘red end’. Note that these plots only include criteria that are unlikely to have been restrained directly during structure refinement. The slider plot provides an easy way for even a non-expert to compare and understand the quality of a PDB entry without having to know the detailed meaning of the individual quality metrics (or their values) used in the plot.

The validation pipeline is now an integral part of the OneDep deposition and annotation system (Young *et al.*, 2017 ▸), and is also made available as a stand-alone server and as an application programming interface (API) so that it can be used during structure determination and refinement. At the time of deposition, the OneDep system provides a preliminary VR to help depositors to address any issues raised before the structure and data are submitted to the archive. This speeds up the process of deposition and annotation and helps to improve the quality of the structures in the archive. The OneDep system produces an official VR after the biocuration of a newly deposited entry has been completed. It is strongly recommended (and for some journals mandatory) that this report is made available to editors and referees as a supporting document with any manuscript that describes a new structure. The VRs are also made public alongside the entries in the PDB archive once the corresponding entries have been publicly released. The archive-wide statistics and the validation-pipeline software are updated and the reports recalculated once a year so that the archive contains up-to-date VRs calculated using the latest version of the validation pipeline and associated reference data.

The VRs were designed with the following goals (Read *et al.*, 2011 ▸).

(i) To apply widely accepted validation metrics with the goal of stimulating their adoption by the depositor and user communities.
(ii) To highlight any major issues concerning the quality of the data and the model at the time of deposition and annotation so that the depositors can fix issues, resulting in improved data quality.
(iii) To help nonspecialist users and referees assess the quality of the coordinate model and supporting experimental data presented in a PDB entry or manuscript.
(iv) To help users identify an appropriate PDB entry or set of PDB entries based on quality assessment for use in their own studies.

To ensure that the validation information is available widely, the wwPDB validation pipeline makes the quality-assessment information available in different formats: a summary and a detailed report presented in PDF format are intended for human readers, whereas the raw information, including validation information for individual residues, is made available in machine-readable XML format. The wwPDB partner sites also make this information available from their web pages. The model-building software *Coot* (Emsley *et al.*, 2010 ▸) uses the per-residue information available in the XML files to display validation information for released PDB entries. The quality information is further used by three-dimensional viewers such as *JSmol* (http://wiki.jmol.org/index.php/JSmol), *LiteMol* (Sehnal *et al.*, 2017 ▸) and *PyMOL* (Schrödinger; https://www.pymol.org/), thus providing multiple ways to access validation data.

A full description of the different sections of the VR has recently been published (Gore *et al.*, 2017 ▸). Here, we describe the most important summary sections that facilitate quality assessment by both expert and nonspecialist users.

The VR summarizes the quality of a structure using the slider plot discussed above. The slider plot includes metrics that assess the geometric quality of the structure: percentile scores for clashscore as well as Ramachandran, side-chain conformation and ‘RNA backbone’ outlier percentages. For structures determined using X-ray crystallography, the percentile score for the value of *R*_free_ (the free *R* value; Brünger, 1992 ▸) and the RSRZ (the real-space *R*-value *Z*-score; Kleywegt *et al.*, 2004 ▸) outlier percentage highlight the agreement between the coordinate model and experimental data in reciprocal and real space, respectively. The overview section also summarizes the validation information for individual polymeric molecules (protein, DNA and RNA chains), highlighting in green, yellow, orange and red the percentage of residues that have one or more outliers for no, one, two or three or more model geometry quality criteria. An additional red bar shows the percentage of residues with RSRZ outliers, *i.e.* residues that show a poorer than expected fit to the electron density. This is followed by a table highlighting outliers for small-molecule ligands, which contains information about any issues with chirality when compared with the standard wwPDB Chemical Component Dictionary (CCD; Westbrook *et al.*, 2015 ▸), outliers in geometry as highlighted using *Mogul* analysis, close contacts and issues with experimental evidence: electron density in the case of X-ray crystallographic structures, as assessed by local ligand density fit (LLDF) analysis (for a detailed examination of ligand validation, see Smart *et al.*, 2018 ▸). The residue-level details concerning outliers are provided in section 3 of the VR, which contains a residue-level plot for each polymeric chain. The colour coding described above is also used in this visualization, thus allowing the identification of individual residues that have outliers. A red dot above a residue indicates a poor fit to the electron density (experimental evidence) for that particular residue.

The existing wwPDB validation pipeline provides comprehensive validation of protein molecules and X-ray experimental data. The second meeting of the X-ray VTF (in October 2015) identified many possible improvements for carbohydrate assessment (Lütteke & von der Lieth, 2004 ▸; Agirre *et al.*, 2015 ▸), as the present reports are restricted to geometric assessment of individual monomers of carbo­hydrate oligomers using *Mogul* (Bruno *et al.*, 2004 ▸). It should be noted that the VR provides a limited set of information on a given structural deposition and it is important to take into account information provided in the publication associated with the entry (if one is available), which will often detail the biological inferences and conclusions that have been drawn from the deposition, possibly in the context of a comparison with other, related structures.

VRs have proven to be a useful tool for journal editors and referees in assessing the quality of the structures described in manuscripts during the review process. As a result, many journals (for example *Acta Crystallographica Section D* and *Section F*, *Nature*, *The FEBS Journal*, *The Journal of Bio­logical Chemistry*, *The Journal of Immunology*, *eLife*, *Angewandte Chemie International Edition*, *Cell*, *Molecular Cell* and *Structure*) are now requesting the submission of VRs alongside manuscripts. The wwPDB partners and the structural biology community would like to see VRs adopted by all journals and their provision made mandatory at the time of manuscript submission if the manuscript describes one or more macromolecular structures. The introduction of VRs for X-ray, NMR and 3DEM structures has fulfilled the goals of wider acceptance of the recommended validation metrics, improved data quality at the time of deposition and helped reviewers, readers and structure users to assess the quality of coordinate models and experimental data.

## 2. Using validation information   

The information in the PDB is used by experts in the structural biology and bioinformatics communities as well as by nonspecialists in the wider life-science community. This is evident from the number of users accessing PDB information from https://pdbe.org, one of the wwPDB partner websites. With more than 1.5 million datafile downloads every day and ∼400 million web accesses annually, it is important to recognise that there are many nonspecialist users with limited knowledge of structural biology whose needs are distinct from those of expert users. Accordingly, the PDBe website has been designed to address the needs of users with a wide variety of expertise (Mir *et al.*, 2018 ▸).

The number of unique UniProt accessions in the PDB is over 43 400 (http://www.ebi.ac.uk/pdbe/docs/sifts/statistics.html), so that there are often multiple PDB entries for a given macromolecule. The resolution and conventional *R* value for structures determined using X-ray crystallography have traditionally been used to select ‘best’ representative structures. The availability of validation reports for all entries in the PDB makes it possible to compare and rank multiple entries containing the same macromolecule using information on structural quality, and thus helps users to select the most suitable entry for their specific purpose.

Fig. 1 ▸ shows the slider plots for two PDB entries for dibenzothiophene desulfurization enzyme B (Lee *et al.*, 2006 ▸). The PDB entries are the structures of the wild-type enzyme (PDB entry 2de2) and of a mutant in complex with a substrate analogue (PDB entry 2de4). The structures are both at 1.8 Å resolution but are in different space groups: PDB entry 2de2 is in *P*2_1_2_1_2_1_ with a single copy of the enzyme in the asymmetric unit, whereas PDB entry 2de4 is in *C*121 with twofold NCS. The two entries have comparable *R* values (0.18 for the wild-type and 0.17 for the mutant structure) and identical values of 0.20 for *R*_free_. The slider plots reveal that the quality of the two entries nevertheless differs considerably based on Ramachandran outliers, clashscore and RSRZ outliers. This and many other cases show that the use of resolution and *R* value may not be sensitive enough to identify the ‘best’ representative structure and that combining the scores for validation metrics and resolution could lead to an improved ranking of PDB entries.

It should be noted that the task of ranking entries is complex and depends on the context of what the user is trying to achieve; for example, identifying the ‘best’ overall structure for a given macromolecule in a set of search results is a very different task to identifying the structure with the ‘best’ binding site for a given ligand. In an accompanying publication (Smart *et al.*, 2018 ▸) we specifically examine ligand validation and the relevant metrics, showing that further work is required to make the latter more reliable.

The PDBe search interface implements functionality for grouping all PDB entries for the same macromolecule and ranking these based on a combined quality metric that takes into account both the resolution and validation metrics for each entry, as described previously (Velankar *et al.*, 2016 ▸),
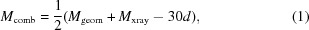
where the combined geometry quality metric *M*_geom_ is the harmonic mean of the percentile scores for *MolProbity* geometric validation metrics of Ramachandran outliers, amino-acid side-chain rotamer outliers, clashscore and RNA suiteness (Richardson *et al.*, 2008 ▸),
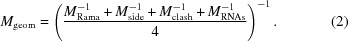



 The combined X-ray data quality metric is the harmonic mean of the percentile scores for *R*_free_ (Brünger, 1992 ▸), as calculated by the *DCC* program (Yang *et al.*, 2016 ▸), and electron-density outliers assessed using RSRZ (Kleywegt *et al.*, 2004 ▸), 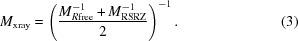
For entries where no experimental data is available, *M*_xray_ is zero.

The last term in (1) takes into account the resolution of a structure by subtracting 30 times *d*, the diffraction resolution limit in Å, from the sum of the two components above. The value of 30 is a somewhat subjective choice that was arrived at by manual analysis of ranking a number of sets of multiple entries.

The reason the overall quality score uses harmonic means instead of arithmetic means is to ensure that an entry with a bad percentile score for any one of the validation metrics is lowered in rank, *i.e.* the overall quality score is pulled towards lower values and cannot be compensated by an excellent score for another validation metric, as would be the case if the arithmetic mean were used.

**Figure 2:**
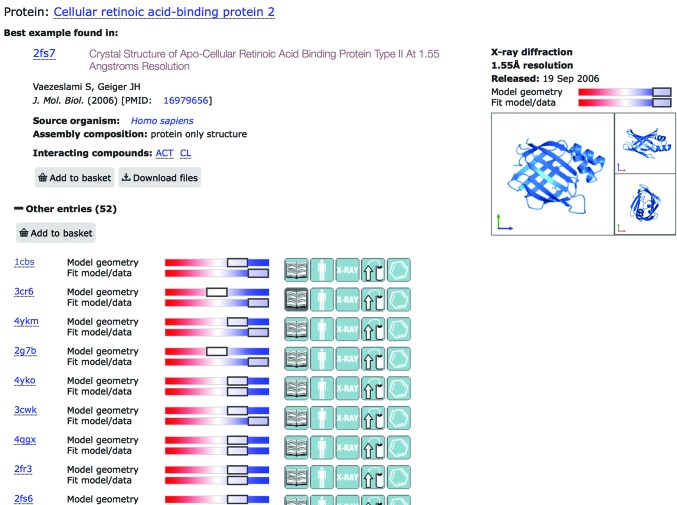
The use of combined validation metrics in the PDBe search facility to rank the available structures of human cellular retinoic acid-binding protein type 2.

An example of using the overall quality score is shown in Fig. 2 ▸. A search for cellular retinoic acid-binding protein type 2 produces a page where the 1.55 Å resolution structure in PDB entry 2fz7 (Vaezeslami *et al.*, 2006 ▸) is presented as the top hit because of its good validation metrics, in preference to a higher resolution structure that has worse geometry scores.

The validation information also includes percentile scores for individual polymeric chains, which makes it possible to rank individual chains in a given entry or to rank different chains that represent the same macromolecule from different entries. Ranking smaller regions, such as residues making up a sequence domain from the Pfam database (Finn *et al.*, 2016 ▸) or a structure domain from the CATH database (Sillitoe *et al.*, 2015 ▸), is more involved because absolute and relative percentile scores for such regions have to be recalculated during the scoring. Another complication that needs to be considered when ranking individual chains or domains is the possibility that the modelled part of a structure may not cover the complete protein molecule or sequence domain. In the case of X-ray crystallographic structures the ranking is further complicated by the common practice of not modelling regions of the molecule that lack clear and unambiguous electron density. Ranking of binding sites is even more complicated owing to the lack of validation metrics that can be compared across different structures. We are currently working on developing methods to be able to rank Pfam domains, and preliminary analysis (results not shown) shows that the overall quality of the entire parent structure (which may include multiple chains and different macromolecules) is not necessarily the most suitable indicator for ranking smaller regions. Instead, the analysis shows that using the individual absolute percentile scores for a particular chain and taking into account the percentage outliers for different validation metrics for a given smaller region (for example covering a certain structure or sequence domain) may provide a better way of ranking such smaller regions. The region-specific quality score in these cases should also take into account the ‘completeness’ of the sequence or structure domain, and in the case of individual chains it takes into account the ‘completeness’ of protein models compared with the sequence in UniProt (The UniProt Consortium, 2017 ▸).

The use of overall and region-specific quality scores can be demonstrated using the 47 PDB entries containing the protein elongin-B, which contains the ‘Ubiquitin’ Pfam domain (accession PF000250). PDB entry 5t35 (Gadd *et al.*, 2017 ▸), although not the highest resolution structure of elongin-B, has better geometric scores and better scores for the fit between the model and experimental data than the other 46 PDB entries, resulting in it being ranked as the ‘best’ entry based on the ‘overall quality’ score (http://www.ebi.ac.uk/pdbe/entry/search/index?pfam_name:ubiquitin&view=macromolecules&molecule_name:%22Elongin-B%22). When ranking only the Pfam domain, which covers residues 4–67 of UniProt accession Q15370, using a region-specific quality score (currently under development), PDB entry 4b9k (Buckley *et al.*, 2012 ▸) is identified as the best example of the ‘Ubiquitin’ Pfam domain in the PDB. This is because the geometric validation metrics for the residues in the region covered by the ‘Ubiquitin’ Pfam domain show that PDB entry 5t35 has geometric outliers for residue 19 (http://www.ebi.ac.uk/pdbe/entry/pdb/5t35/protein/2) in chain *B*. Chain *F* does not show any outliers and is ranked higher than chain *B*. PDB entry 4b9k is a higher resolution structure (2.0 Å compared with 2.7 Å for PDB entry 5t35) and does not show any outliers in the region of the Pfam domain (http://www.ebi.ac.uk/pdbe/entry/pdb/4b9k/protein/1) in chain *G*, and hence should be listed at the top of the web-search results for the ‘Ubiquitin’ domain in elongin-B. More work is required to finalize the domain-scoring methodology.

## 3. ValTrends^DB^   

ValTrends^DB^ (Svobodova Varekova *et al.*, 2017 ▸) is an annually updated database that contains the results of an analysis of various properties of biomacromolecular structures and ligands and their relationships. This analysis was carried out to discover interesting and scientifically relevant trends between structure-quality criteria and geometrical, experimental and biochemical properties. The input data, from which all properties were computed, were acquired from two sources. Structure metadata, as well as structure-quality data and some of the ligand-quality data, were obtained from the Protein Data Bank in Europe (PDBe) database. The Validator^DB^ database (Sehnal *et al.*, 2015 ▸) provides additional information about the agreement of ligand geometry with the wwPDB CCD; for example, highlighting ligands with nomenclature errors or chiral inversions. The version of ValTrends^DB^ used in this work is based on data from 17 February 2017 and contains 88 properties computed for 112 766 PDB entries (only structures obtained using X-ray crystallography have been included). From these properties, 1852 meaningful and scientifically interesting property pairs were manually selected from all the possible pairs. These pairs were then assessed for the existence of a trend using interval analysis. Application of this statistical processing method was necessary since the distribution of most property values was heavily skewed. The strength of a relationship was quantified using Spearman’s rank correlation coefficient, which provides a statistical measure of whether two variables have a monotonic relationship.

All of the results of the analysis are freely available on the ValTrends^DB^ website (http://ncbr.muni.cz/ValTrendsDB). Users can view precomputed plots to see the trends (or the lack thereof) that were discovered during the analysis. It is also possible to render plots of property-pair relationships using various custom settings. Users can view the distribution of every property, as well as download the source data for the database, and a table with Spearman’s coefficients for all studied relationships.

**Figure 3:**
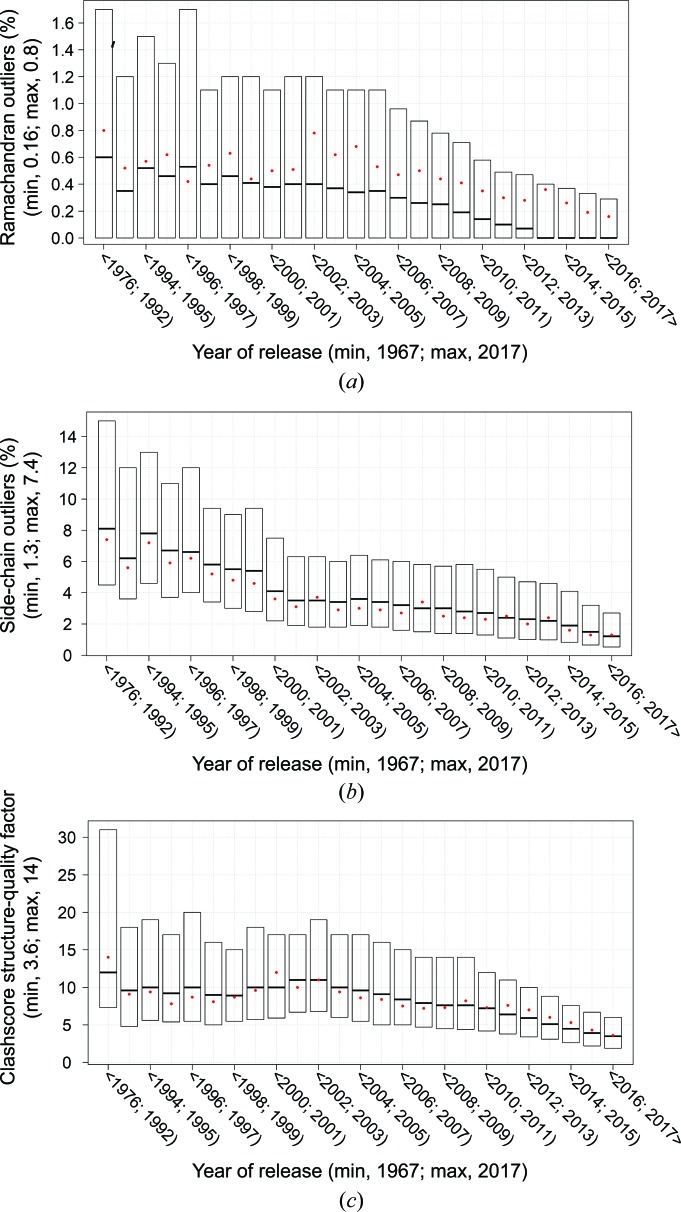
ValTrends^DB^ plots showing how the overall validation metrics for protein geometry, as reported in wwPDB VRs, have improved with time. (*a*) Percentage Ramachandran outliers as assessed by *MolProbity* (Chen *et al.*, 2010 ▸). (*b*) Percentage side-chain rotamer outliers as assessed by *MolProbity*. (*c*) *MolProbity* all-atom clashscore. Only structures determined by X-ray crystallography were included in the analysis. In each case the box plot shows the inter-quartile range. The red dot marks the weighted arithmetic mean as explained in Appendix *A* [App appa].

**Figure 4:**
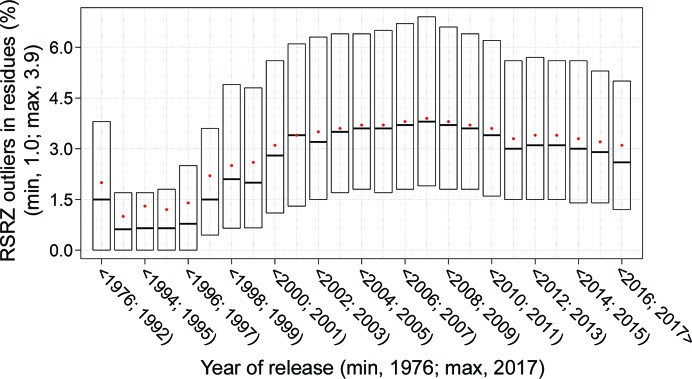
ValTrends^DB^ plot showing how the percentage RSRZ electron-density-fit outliers metric (Kleywegt *et al.*, 2004 ▸) varies with deposition date.

ValTrends^DB^ has been used to assess how protein validation metrics vary with time, following on from the work of Kleywegt & Jones (2002 ▸). Fig. 3 ▸ shows how metrics that assess protein geometry have progressively improved with time. In contrast, Fig. 4 ▸ shows that the proportion of residues judged to be electron-density-fit outliers behaves very differently. Structures released prior to 1997 have low values, with median values below 2%, and there is a progressive increase to a median value of around 4% in 2007. This might be owing to a number of factors such as the large increase in the number of structures deposited in the late 1990s or that larger or more difficult structures were being solved. Following the increase, there has been a gradual decrease to around 3% median outliers that could be owing to improved refinement and building protocols (Emsley *et al.*, 2010 ▸). Note that the proportion of density-fit outliers is not approaching zero, unlike the geometry outliers, as there are normally regions in the typical electron-density map that are difficult to build a model into, particularly at medium and low resolution.

It is interesting to compare the long-term trends with the observations of Shao *et al.* (2017 ▸), who used a different methodology and compared ‘legacy’ depositions from 2012 to 2013 with more recent depositions from 2014 to 2015. Shao and coworkers noted improvements in all of the metrics and attributed the effects to the introduction of the VR during this time. It can be noted that the improvements appear to be consistent with a continuation of long-term trends that makes attribution difficult.

## 4. Discussion and conclusions   

The PDB, one of the first open-access data resources in the life-science field, was established as an archive of macromolecular structure data in 1971 with the participation of the structural biology community. The archive is now managed by the wwPDB, an international collaboration of four partner sites: RCSB PDB and BMRB in the USA, PDBj in Japan and PDBe in Europe.

Early on, the PDB archive established a process for the validation of macromolecular structure data by implementing geometric validation of protein structures and supporting the deposition of experimental data in the form of structure-factor amplitudes or intensities for X-ray crystallographic structures. The wwPDB partners have established VTFs that have recommended community-accepted validation criteria for model coordinates and specific metrics for assessing experimental data for each experiment type. Based on the recommendations of the VTFs, the wwPDB has implemented a validation pipeline that supports the efforts by the structural biology community to improve the quality of the structure models deposited in the PDB. The validation pipeline integrates community-accepted standard software for assessing the quality of macromolecular models and experimental data. The validation pipeline also provides, for the first time, a method for assessing the geometry of bound molecules using the CCDC *Mogul* software. The validation reports have adopted the slider-plot visualization recommended by the X-ray VTF, which provides an easy way for even a non-expert to compare and understand the quality of a PDB entry without having to know the detailed meaning of the individual quality metrics provided in the slider plots.

Analysis of structure-quality information for the PDB archive shows that the introduction of VRs has coincided with improvements in the quality of the structures deposited in the PDB. This is probably owing to a combination of factors, including (i) increased awareness of the importance of validation in the depositor community, (ii) the software-development community providing easy access to validation methods, (iii) the availability of validation reports before and after biocuration, which enables depositors to resolve any issues highlighted in them, (iv) the requirement by an increasing number of journals for submission of the validation reports with manuscripts describing new structures and (v) the availability of validation reports for all legacy structures in the PDB (X-ray, NMR and 3DEM).

Continuous monitoring of trends in the quality of macromolecular structures in the PDB archive can be performed with ValTrends^DB^, which provides statistical analysis of a large number of validation metrics and properties of biomacromolecules and bound ligands. The web interface provides easy access to the statistical analyses through plots that clearly show pairwise relationships between selected properties. Users have access to both the raw data and data from the statistical analysis, both of which will be updated annually.

The PDB archive contains multiple structures of many macromolecules that may have been determined using different experimental methods or in complex with different ligands or under different conditions. With more general biologists accessing PDB data as well as many life-science resources that use PDB information, it is important that users are provided with an easy way to select the structure of their molecule of interest that is most suitable for their needs from a possible abundance of structures available in the archive. Traditionally, resolution and *R* factor have been the two criteria used to select the best representative entry. Our analysis shows that ranking of relevant entries using validation information can help to identify the ‘best’ representative. The PDBe query system implements an overall quality measure to rank PDB entries taking into account the geometric quality, the quality of the fit of the model to the experimental data and the resolution.

It should be noted that the development of VRs, validation metric sliders and combined metrics should not mean that they are the sole criteria of whether a structure has been adequately determined. One danger of VRs is that a ‘clean report’ becomes an aim in itself, as opposed the production of a model that is the most faithful interpretation of the experimental data in the light of the prior knowledge. If a model is slightly adjusted to get rid of validation issues then this is unlikely to produce any meaningful improvement in its real quality; instead, the VR criteria are then used as a ‘refinement target’ and lose their value as independent validation criteria (Kleywegt, 2000 ▸). VRs do not currently include any evaluation of difference density peaks or unmodelled ‘blobs’ of difference density (Emsley *et al.*, 2010 ▸). Hence, it is often possible to cosmetically ‘improve’ a VR, without meaningful improvement of the model, simply by removing parts of the structure that are flagged as outliers, albeit at the cost of a small increase in *R*_free_. Such action obviously does not improve a model in any real sense. More work needs to be performed to develop additional criteria and guidance as to the appropriate action to be taken in response to VR outlier reports.

Although the overall quality serves as a good measure for ranking a set of PDB entries, it does not necessarily provide a good measure for ranking a particular protein chain or part of the chain (binding site, sequence or structure domain), as quality assessment of different chains in a given entry can show variation. The ‘completeness’ of the coordinate model for a particular region of a chain is also an important criterion in selecting the ‘best’ representative for a region of interest from a set of PDB structures. This is particularly important for the many life-science resources that use PDB data and display only a list of PDB codes on their web pages instead of providing a representative structure that is relevant to the information presented. If they use the ranking system described here, these resources could display the ‘best’ representative structure and additional coordinate-based information. The PDBe REST API allows such ranking, and multiple data resources, such as Reactome (Fabregat *et al.*, 2017 ▸), OpenTargets (Koscielny *et al.*, 2017 ▸) and Complex Portal (Meldal *et al.*, 2015 ▸), use this approach to display PDB information on their web interface.

## Appendix A Methodology for computation of the weighted arithmetic mean in the ValTrends^DB^ analysis

The weighted arithmetic mean was enumerated for every interval of every pair of properties in the analysis. It is based on the assignment of a weight to every value in the interval. In the analysis, each interval was split into twn identically sized subintervals. A weight equal to the number of structures in each subinterval was then assigned to each value. By multiplying each value by its weight, we obtained a set of weighted values. Finally, the weighted arithmetic was computed as sum of weighted values divided by the sum of weights.
